# Identification of cognitive impairment in patients with hypertension: a systematic review and critical appraisal of existing models

**DOI:** 10.3389/fmed.2026.1858790

**Published:** 2026-08-10

**Authors:** Yueliang Sun, Jiankang Wang, Fengling Sun, Mingpeng Shi, Xinxin Tian, Hang Shang, Wenxuan Wang, Tianying Chang, Jiajun Zhao, Yongsheng Huang, Yingzi Cui, Jiajuan Guo

**Affiliations:** 1 College of Traditional Chinese Medicine, Changchun University of Chinese Medicine, Changchun, China; 2 Department of Cardiology, Affiliated Hospital of Changchun University of Chinese Medicine, Changchun, China; 3 Orthopedic Center, Affiliated Hospital of Changchun University of Chinese Medicine, Changchun, China; 4 Department of Geriatrics, Affiliated Hospital of Changchun University of Chinese Medicine, Changchun, China; 5 Changchun University of Chinese Medicine Affiliated Hospital, Changchun, China; 6 Academic Affairs Office, Changchun University of Chinese Medicine, Changchun, China

**Keywords:** cognitive impairment, critical appraisal, hypertension, risk prediction, systematic review

## Abstract

**Background:**

Hypertension is strongly associated with cognitive impairment. Existing tools include models designed to identify prevalent cognitive impairment and a smaller number developed to predict future risk. However, their methodological quality, validation status, and reproducibility have not been systematically assessed. We conducted a systematic review and critical appraisal of existing models designed to identify cognitive impairment in patients with hypertension.

**Methods:**

Chinese and English databases were searched from inception to December 23, 2025. Studies that developed, updated, or validated a multivariable model, nomogram, score, or machine-learning tool for cognitive impairment, mild cognitive impairment, cognitive decline, subjective cognitive decline, or dementia among adults with hypertension were included. Data were extracted using CHARMS, and risk of bias was assessed with PROBAST. The synthesis emphasized model purpose, temporality, outcome definition, discrimination, calibration, validation, risk of bias, and reproducibility. Reported c-statistics were summarized descriptively because of substantial heterogeneity in study design, outcome definitions, cognitive assessment tools, and repeated model estimates from the same datasets.

**Results:**

Seventeen studies reporting 25 models were included. Most models were developed in China using cross-sectional data, with cognitive outcomes assessed using varied instruments. Reported AUCs ranged from 0.344 to 0.938; most multivariable models exceeded 0.70. Internal validation was reported in 15 studies, whereas only four conducted external validation and 11 assessed calibration. Sixteen studies were judged at high risk of bias. Reproducibility was limited: four studies provided sufficient information for independent calculation, eight were partially reproducible, and five were insufficiently reported. Common predictors included older age, lower education, depression, obesity, longer hypertension duration, greater hypertension severity, and adverse metabolic indicators.

**Discussion:**

Existing models show some ability to distinguish hypertensive patients with and without cognitive impairment, while evidence supporting future-risk prediction remains limited. Future work should prioritize prospective cohorts, standardized outcome definitions, external validation, calibration, and transparent reporting of equations, intercepts, scoring rules, code, and model objects.

**Systematic review registration:**

https://www.crd.york.ac.uk/PROSPERO/view/CRD420251157136, identifier CRD420251157136.

## 1 Introduction

Hypertension is a leading modifiable risk factor globally for cardiovascular events, kidney disease, and target organ damage. Its escalating prevalence has rendered it a major public health challenge (1). With the acceleration of population aging, hypertension-related cognitive impairment is increasingly becoming a core issue affecting the quality of life and independent living ability of the elderly (2, 3). Mild cognitive impairment (MCI), serving as an intermediate stage between normal aging and dementia, is a crucial window of opportunity for therapeutic intervention (4). Furthermore, hypertension represents a well-established, modifiable, and independent risk factor for this condition (5, 6). Research has demonstrated that hypertension promotes cognitive decline via multiple pathways, leading to a markedly increased risk of MCI and dementia among individuals with hypertension relative to those with normal blood pressure (7, 8).

Hypertension-related cognitive decline is driven by multiple mechanisms, including cerebrovascular dysfunction, small vessel disease, chronic hypoperfusion, and neuroinflammation (9, 10). These processes may impair brain structure and connectivity (11, 12) and, together with interactions with Alzheimer’s disease pathology, accelerate cognitive decline (13, 14). Therefore, early identification of cognitive impairment risk in hypertensive patients is clinically important.

Although the association between hypertension and cognitive impairment is widely recognized, accurately identifying high-risk individuals in clinical practice remains a significant challenge. Currently, there is a lack of a rigorously validated model specifically designed either to identify prevalent cognitive impairment or to predict incident cognitive decline in the hypertensive population (15). Many existing models have significant methodological limitations. For instance, most are based on cross-sectional study designs, where the outcome may have already occurred at the time the predictors are measured, posing a potential risk of reverse causality (16, 17). Moreover, the lack of a consistent variable selection strategy, insufficient reporting on model calibration, and the absence of external validation severely hinder their clinical generalizability and practical utility (18, 19).

This review systematically maps and critically appraises existing models for identifying or predicting cognitive impairment in patients with hypertension. It examines study designs, outcome definitions, diagnostic criteria for hypertension, modeling methods, validation strategies, calibration assessment, risk of bias, and model reproducibility. Particular attention is given to whether sufficient information is reported to enable independent external validation. By clarifying the current state of research and identifying key methodological limitations, this work is expected to provide valuable guidance for the future development of robust and clinically applicable prediction models, ultimately contributing to more precise management of cognitive health in patients with hypertension.

## 2 Methods

### 2.1 Study design

The present study adhered to the PRISMA guidelines (20) and the CHARMS statement (21). The study protocol was registered in the PROSPERO database (Registration ID: CRD420251157136).

### 2.2 Establishment of the problem

This study adopted the PICOTS framework endorsed by the Cochrane Prognosis Methods Group to formulate the evidence-based question. The study population (P) consisted of patients with hypertension. The index tool (I) was any multivariable clinical prediction model, risk score, nomogram, or machine-learning model designed to identify cognitive impairment. There was no comparator (C). Outcomes (O) included cognitive impairment, MCI, cognitive decline, subjective cognitive decline, or dementia, as defined by each original study. Each outcome definition, cognitive scale, and diagnostic threshold was extracted separately because these constructs reflect different clinical states and measurement approaches. Timing (T) referred to cross-sectional identification at assessment or prospective prediction during follow-up, depending on study design. Settings (S) included hospital, community, database, and other clinical or public-health settings.

### 2.3 Search strategy

A comprehensive literature search on risk prediction models for cognitive impairment in hypertensive patients was conducted using multiple databases, including CNKI, Wanfang, VIP, SinoMed, PubMed, Embase, the Cochrane Library, and Web of Science. The search was limited to literature published up to December 23, 2025, and the specific search terms and strategies used for both Chinese and English databases are detailed in Supplementary Additional file 1.

### 2.4 Inclusion and exclusion criteria

Inclusion criteria: (1) Adults aged 18 years or older with hypertension; (2) Development, updating, or validation of a multivariable model, nomogram, risk score, or machine-learning tool for identifying or predicting cognitive impairment; (3) Cognitive outcomes assessed using a recognized cognitive scale, questionnaire, or clinical diagnosis; (4) Reporting of at least one model-performance measure, model presentation format, or predictor set; (5) Publication in English or Chinese.

Exclusion criteria: (1) Case reports, reviews, commentaries, editorials, conference abstracts, or other non-original studies; (2) Studies not involving patients with hypertension or not reporting separate results for hypertensive patients; (3) Studies that did not establish or validate any predictive model; (4) Animal or other non-clinical studies.

### 2.5 Study selection and data extraction

The references were managed using EndNote 21. Two investigators (SYL and WJK) independently screened the studies against the pre-defined inclusion and exclusion criteria by examining titles, abstracts, and full-text articles. Dissertations were considered eligible only if they met the same inclusion criteria as journal articles. Any discrepancies were resolved by consulting a third reviewer (GJJ). Standardized data extraction forms were created following the CHARMS guideline (21). Data extraction was performed independently by two investigators (SYL and SMP), with any disagreements discussed with a third researcher (GJJ). The data extraction covered the following:

General study characteristics, such as first author, year of publication, country, study type, timeline, total patient number (male/female; cognitively normal/cognitive impairment), age, assessment instruments, study population, hypertension diagnostic criteria, and sample origin (Table 1).

**TABLE 1:** General characteristics of the included risk models.

First author (year)	Country	Type of study	Timeline	No. of participants (male/female)	No. of participants (Nomal/NCD)	Years (mean ± SD) or (min,max)	Assessment tools	Populations	Diagnostic criteria for HTN	Sample sources
Liu (25)	China	Cross-sectional	October 2020 to February 2021	300 (180/120)	300 (225/75)	78.84 ± 11.53	MMSE	Older HTN patients (≥60 years)	①	Cadre Ward, The First Hospital of Jilin University
Wei (29)	China	Prospective cohort	2018 to January 2021	232 (99/133)	232 (81/151)	68.0 (65.0,72.0)	MoCA	Older HTN patients (≥60 years)	④	Qiaohua Community and Xing’an South Road Community, Hohhot City
Long (26)	China	Cross-sectional	July 2021 to November 2022	173 (89/84)	173 (85/88)	Normal:64.962 ± 8.803 Mildly NCD:68.77 ± 6.791 Moderately NCD:78.241 ± 7.614 Severely NCD:87.846 ± 5.145	MMSE MoCA	Older HTN patients (≥60 years)	①	General Medical Department, Affiliated Hospital of Chengde Medical College
Wang et al. (28)	China	Cross-sectional	May to November 2021	182 (120/62)	182 (58/124)	Normal:54 (47,66) NCD:60.5 (54,67)	MMSE MoCA	Hospitalized HTN patients	④	Department of Cardiology, The First Affiliated Hospital of Army Medical University
Lu et al. (35)	China	Cross-sectional	D: April–July 2020; V: August–October 2020.	D: 345 (172/173) V: 146 (78/68)	D:345 (148/197) V:146 (65/81)	D:73.61 ± 7.02 V:73.20 ± 6.74	MMSE MoCA	Older HTN patients (≥65 years)	⑤	Xixiangtang District (D) and Qingxiu District (V), Nanning City, China
Xia et al. (37)	China	Cross-sectional	May 2022 to December 2022	total:733 (359/374) D:513 (236/277) V:220 (123/97)	total:733 (611/122)	66.37 ± 10.88	MMSE	Hospitalized HTN patients (30–85 years)	②	8 hospitals in 5 prefecture-level cities, Shandong Province
Feng et al. (24)	China	Cross-sectional	January 2020 to December 2021	206 (103/103)	206 (138/68)	Normal: 71 (65,75) MCI: 71 (65,74)	MoCA	Older HTN patients (≥65 years)	④	Department of Geriatrics, The First Affiliated Hospital of Jinzhou Medical University
Ma et al. (27)	China	Cross-sectional	September to November 2011	509 (198/311)	509 (412/97)	66.3 ± 6.4	MMSE	Community HTN Patients (≥55 years)	①	5 communities in Yinchuan City and Wuzhong City, Ningxia Hui Autonomous Region
Zhang et al. (32)	China	Cross-sectional	February 2022 to October 2022	137 (71/66)	137 (87/50)	Normal:67.48 ± 6.33 NCD:70.88 ± 8.88	MMSE	Older HTN patients (≥65 years)	④	General Hospital of Ningxia Medical University
Li et al., (36)	China (CHARLS and CLHLS databases)	Cross-sectional	2018	D:1121 (230/891) V:4016 (1717/2299)	D:1121 (136/985) V:4016 (3521/495)	CHARLS:63.3 ± 10.3 CLHLS:86.4 ± 10.6	MMSE,CSI-D	Community HTN patients	-	CHARLS and CLHLS databases
Zhong et al. (33)	China	Cross-sectional	January 2020 to March 2023	502 (232/270)	502 (398/104)	Normal:71.41 ± 6.97 NCD:76.03 ± 7.29	MMSE	Older HTN patients (≥60 years)	④	The Affiliated Hospital of Shandong University of Traditional Chinese Medicine and 10 other hospitals
Zuo and Yang (39)	USA (NHANES database)	Cross-sectional	2011–2014	total:1517 (765/752) D:1065 (534/531) V:452 (218/234)	1517 (1138/379)	69 (64,76)	CERAD,AFT,DSST	Older HTN patients (≥60 years)	②	NHANES database (National Health and Nutrition Examination Survey, USA)
Jiang et al., (34)	China	Cross-sectional	March 2023 to February 2024	total: 1098 D: 783 (374/409) V:315	total: 1098 D:783 (420/363) V:315 (182/133)	≥60	SCD-9	Older HTN patients (≥60 years)	④	Department of Cardiology, 2 tertiary general hospitals in Jinzhou City, Liaoning Province
Zhong et al. (38)	China	Cross-sectional	May 2022 to February 2024.	757 (371/386)	757 (625/132)	67.11 ± 11.47	MMSE	HTN patients	②	4 prefecture-level hospitals in Yantai, Jinan, Weifang, and Dongying
Dong (23)	China	Cross-sectional	February 2022 to February 2024	405 (239/166)	405 (249/156)	≥65	MMSE	Older HTN patients (≥65 years)	④	Outpatient Clinic, Nanjing Central Hospital
Yang et al. (30)	China (CHARLS Database)	Cross-sectional	D: 2011–2020; V: January 2024 to December 2024	total:6024 D:4167 (2096/2071) V:1457	D:4167 (3499/668) V:1457	Normal:60.31 ± 9.08 NCD:65.64 ± 9.51	CHARLS Cognitive Tests	HTN patients (≥45 years)	–	D: CHARLS database; V: Chongqing Traditional Chinese Medicine Hospital
Yin (31)	China	Cross-sectional	November 2023 to March 2025	611 (322/289)	611 (475/136)	65.02 ± 10.446	MMSE	HTN patients	⑥	Community health centers/villages in Gannan Tibetan Autonomous Prefecture

D, modeling set; V, validation set; NCD, neurocognitive disorders (normal refers to only suffering from hypertension, NCD refers to hypertension combined with neurocognitive disorders); MoCA, Montreal Cognitive Assessment; MMSE, Mini-Mental Status Examination; CSI-D, Community Screening Instrument for Dementia; CERAD, Consortium to Establish a Registry for Alzheimer’s Disease; AFT, Animal Fluency Test; DSST, Digit Symbol Substitution Test; SCD-9, Subjective Cognitive Decline Questionnaire; HTN, hypertension; ①, Chinese Guidelines for the Prevention and Treatment of Hypertension 2010; ②, 2014 Evidence-Based Guideline for the Management of High Blood Pressure in Adults, Report From the Panel Members Appointed to the Eighth Joint National Committee (JNC 8); ③, ACC/AHA 2017 Guidelines; ④, Chinese Guidelines for the Prevention and Treatment of -Hypertension 2018; ⑤ NICE guideline [NG136] Hypertension in adults, diagnosis and management (2019); ⑥, Chinese Guidelines for the Prevention and Treatment of Hypertension (2024 Revision).

Details related to the prediction models, which encompassed the modeling technique, number of candidate variables, events per variable (EPV), variable screening method, treatment of continuous variables, model validation approach, model performance [e.g., area under the receiver operating characteristic curve (AUC) values, calibration methods], model presentation format, and TRIPOD statement adherence level (Table 2) (16, 18).

**TABLE 2:** Details related to the prediction models.

Study	Methods	Candidate variables (number)	EPV	Screening variable methods	Continuous variable handling methods	Model validation methods	AUC (95% CI)	Calibration method	Model presentation	TRIPOD levels^a^
Liu (25)	LR	34	15.00	Single-factor, multi-factor analysis	Maintain continuity	Internal validation (ROC curve analysis)	DSI:0.642 (−) Age:0.630 (−) TC: 0.607 (−) Activities:0.625 (−) Albumin:0.618 (−)	–	–	1a
Wei (29)	LR	26	50.33	Single-factor, multi-factor analysis	Transfer to categorical variables	Internal validation (bootstrap)	SBPV:0.740 (−) DBPV:0.748 (−) PPV:0.740 (−)	H-L: *p* > 0.05	Nomogram	1b
Long (26)	LR	23	17.60	Single-factor, multi-factor analysis	Maintain continuity	NR	sNFL:0.70 (−)	–	–	1a
Wang et al. (28)	LR	15	24.80	Single-factor, multi-factor analysis	Maintain continuity	Internal validation (bootstrap)	D:0.844 (0.783–0.893)	Calibration curve	Nomogram	1b
Lu et al. (35)	LR	28	25.27	Single-factor, multi-factor analysis	Transfer to categorical variables	Internal validation (ROC curve analysis) External validation (geographical validation)	D:0.795 (0.749–0.841) V2:0.765 (0.688–0.842)	H-L: *p* = 0.703 (D)/0.234 (V) Calibration curve	Nomogram	2b
Xia et al. (37)	XGBoost LR GNB	42	30.50	LASSO	Maintain continuity	Internal validation (5-fold cross-validation)	V1(LR):0.83 (0.79–0.87) V1(XGB):0.88 (0.85–0.91) V1(GNB):0.82 (0.78–0.86)	Calibration curve	Nomogram	2a
Feng et al., (24)	LR	23	17.00	Single-factor, multi-factor analysis	Maintain continuity	Internal validation (ROC curve analysis)	Hcy:0.870 (0.808–0.931) 25(OH)D:0.834 (0.774–0.895) Uric acid:0.683 (0.599–0.766) D:0.921 (0.881–0.961)	H-L: *p* = 0.950	Formula	1a
Ma et al. (27)	LR	17	16.17	Single-factor, multi-factor analysis	Transfer to categorical variables	Internal validation (ROC curve analysis)	D:0.72 (0.65,0.78)	–	–	1a
Zhang et al. (32)	LR	9	10.00	Single-factor, multi-factor analysis	Maintain continuity	Internal validation (ROC curve analysis)	Education years: 0.365 (0.268,0.462) HBP duration: 0.770 (0.693,0.848) SUA: 0.741 (0.656, 0.826) TC: 0.583 (0.486,0.820) FBG: 0.344 (0.234,0.434) D: 0.907 (0.864,0.960)	–	Formula	1a
Li et al. (36)	GLM	21	185.00	LASSO	Transfer to categorical variables	Internal validation (14-fold cross-validation) External validation (geographical validation)	D:0.777 (−) V1:0.785 (−) V2:0.782 (−)	H-L: *p* = 0.346(D)/0.626 (V) Calibration curve	Nomogram Formula	3
Zhong et al. (33)	LR XGBoost AdaBoost SVM GNB MLP	22	13.00	LASSO	Maintain continuity	Internal validation (10-fold cross-validation)	D(XGBoost):0.938 (0.911–0.965) V1(XGBoost):0.878 (0.756–0.987) D(LR):0.872 (0.830–0.914) V1(LR):0.862 (0.734–0.976) D(AdaBoost):0.911 (0.879–0.944) V1(AdaBoost):0.875 (0.760–0.983) D(SVM):0.818 (0.767–0.870) V1(SVM):0.821 (0.674–0.963) D(GNB):0.865 (0.824–0.906) V1(GNB):0.867 (0.753–0.973) D(MLP):0.583 (0.519–0.647) V1(MLP):0.535 (0.350–0.720)	–	Nomogram	2a
Zuo and Yang (39)	LR	19	54.14	1:LASSO 2:Single-factor, multi-factor analysis	Maintain continuity	Internal validation (10-fold cross-validation)	D:0.802 (0.773–0.831) V1:0.756 (0.707–0.805)	Calibration curve	Nomogram	2a
Jiang et al. (34)	LR	20	62.00	Single-factor, multi-factor analysis	Transfer to categorical variables	Internal validation (bootstrap) External validation (temporal validation)	D:0.877 (0.853–0.901) V2:0.813 (0.767–0.859)	H-L: *p* = 0.692 Calibration curve	Nomogram	2b
Zhong et al. (38)	XGBoost LR AdaBoost GNB SVM	18	22.00	LASSO	Maintain continuity	Internal validation (5-fold cross-validation)	D(XGBoost):0.893 (0.856–0.931) V1(XGBoost):0.770 (0.706–0.834) D(LR):0.803 (0.750–0.856) V1(LR):0.788 (0.731–0.844) D(AdaBoost):0.854 (0.812–0.896) V1(AdaBoost):0.771 (0.714–0.827) D(GNB):0.778 (0.715–0.840) V1(GNB):0.749 (0.682–0.816) D(SVM):0.645 (0.575–0.716) V1(SVM):0.709 (0.645–0.773)	Calibration curve	–	2a
Dong, (23)	LR	20	15.60	Single-factor, multi-factor analysis	Transfer to categorical variables	NR	D:0.798 (0.710–0.886)	–	Risk Score	1a
Yang et al. (30)	LR	17	83.50	LASSO	Transfer to categorical variables	Internal validation (10-fold cross-validation) External validation (temporal validation)	D:0.814 (0.802–0.826) V1:0.817 (0.788–0.846) V2:0.725 (0.699–0.752)	Calibration curve	Nomogram	3
Yin, (31)	LR	12	19.43	Single-factor, multi-factor analysis	Transfer to categorical variables	Internal validation (bootstrap)	D:0.881 (0.849–0.912)	H-L: *p* = 0.9694	Nomogram	1b

LR, logistic regression; XGBoost, extreme gradient boosting; GNB, gaussian naive bayes; GLM, generalized linear model; AdaBoost, adaptive boosting; SVM, support vector machines; MLP, multilayer perceptron; EPV, events per variable (indicates the number of events per independent variable); LASSO, least absolute shrinkage and selection operator; ROC, receiver operating characteristic; AUC, area under the ROC curve; DSI, depression status; TC, total cholesterol; SBPV, systolic blood pressure variability; DBPV, diastolic blood pressure variability; PPV, pulse pressure variability; sNFL, serum neurofilament light chain protein;; D, modeling set; V1, internal validation set; V2, external validation set; Hcy, homocysteine; HBP, high blood pressure; SUA, serum uric acid; FBG, fasting blood glucose; H-L, Hosmer-Lemeshow goodness-of-fit test.

*^a^*Transparent Reporting of a multivariable prediction model for Individual Prognosis Or Diagnosis (TRIPOD) levels, 1a = development only; 1b = development and validation using resampling; 2a = random split sample development and validation; 2b = non-random split sample development and validation; 3 = development and validation using separate data; 4 = validation only.

Model reproducibility was assessed explicitly for each included study. We recorded whether the original publication provided regression coefficients, intercepts, complete formulas, scoring rules, total-score-to-risk conversion, nomograms, online calculators, code, model objects, hyperparameters, or sufficient information to permit independent external validation. Models were categorized as having sufficient information when a third party could calculate an individual-level predicted probability without contacting the authors. Models were considered partially reproducible when a nomogram, score, coefficient set without intercept, or online tool allowed approximate use but did not support exact reconstruction. Models were considered insufficiently reproducible when only odds ratios (ORs), AUCs, selected predictors, SHapley Additive exPlanations (SHAP) plots, or variable-importance rankings were reported without a usable prediction rule (Table 3).

**TABLE 3:** Model reproducibility and readiness for independent external validation.

C	Key reproducibility information reported	Readiness	Critical appraisal
Liu, (25)	Predictors/ORs and single-index ROC results; no equation, intercept, score, or nomogram.	Insufficient	Suitable as risk-factor evidence, not a reproducible prediction model.
Wei (29)	Blood-pressure-variability nomograms and internal validation; no precise coefficients or intercepts.	Partial	Approximate validation possible by reading nomograms, but exact probability reconstruction is not possible.
Long (26)	sNFL diagnostic ROC and regression results; no complete model equation.	Insufficient	More consistent with diagnostic-marker analysis than a reproducible prediction model.
Wang et al. (28)	Logistic equation, coefficients, intercept, and nomogram.	Sufficient	Independent calculation of individual risk is possible.
Lu et al. (35)	Regression coefficients/ORs and nomogram with internal and external validation; intercept not clearly reported.	Partial	Approximate nomogram-based validation possible; exact probability reconstruction is limited.
Zhong et al. (37)	Machine-learning predictors, AUCs, SHAP, and nomogram; no code, model object, or hyperparameters.	Insufficient	Reported performance cannot be independently reproduced exactly.
Feng et al. (24)	Complete logistic formula with intercept and coefficients.	Sufficient	Independent calculation and external validation are feasible.
Ma et al. (27)	Regression coefficients and weighted risk value; no intercept or complete probability conversion.	Partial	Relative risk score can be examined, but calibration cannot be reproduced exactly.
Zhang et al. (32)	Complete logistic formula with intercept and coefficients.	Sufficient	Independent risk calculation is feasible.
Li et al. (36)	Generalized linear model formula, intercept, nomogram, and external validation using CLHLS.	Sufficient	One of the most reproducibly reported models.
Zhong et al. (33)	XGBoost model, SHAP, and nomogram; no code, model object, tree structure, or hyperparameters.	Insufficient	Machine-learning model is not independently reproducible.
Zuo (39)	Coefficients and web-based dynamic nomogram; intercept not clearly reported in text.	Partial	Usable through online tool, but offline exact reconstruction is restricted.
Jiang et al., (34)	Nomogram/dynamic nomogram and internal/external validation; complete formula not clearly reported.	Partial	Approximate clinical use possible; exact model reconstruction remains limited.
Zhong et al. (38)	XGBoost model with environmental predictors, SHAP, and DCA; no code or trained model object.	Insufficient	Performance claims require independent validation and reproducible model sharing.
Dong (23)	Integer risk-score rule and cut-off; no probability equation or intercept.	Partial	External validation of risk strata is possible, but absolute risk calibration is not.
Yang et al. (30)	Regression coefficients and nomogram with external validation; intercept not clearly reported.	Partial	Strong validation design, but exact independent probability calculation is limited.
Yin (31)	Regression coefficients and nomogram; no complete formula or intercept.	Partial	Approximate nomogram validation is possible; exact reconstruction is limited.

### 2.6 Quality assessment

This study utilized the Prediction model Risk Of Bias ASsessment Tool (PROBAST) to evaluate the quality of the included studies and the potential risk of bias in the prediction models (17). The PROBAST assesses the risk of bias across four domains: participants, predictors, outcome, and analysis. It comprises 20 signaling questions, which are answered as “yes/probably yes,” “no/probably no,” or “no information.” A risk of bias was judged as “low” if all signaling questions were answered “yes” or “probably yes.” Conversely, the risk was judged as “high” if any question was answered “no” or “probably no.” If some questions were rated as “no information” but all others were “yes” or “probably yes,” the overall risk of bias was categorized as “unclear.” The overall judgments for both risk of bias and concerns regarding applicability were classified as “low,” “high,” or “unclear” (Table 4). Two investigators (SYL and TXX) independently evaluated the study quality, the risk of bias in the models, and concerns regarding applicability. Any discrepancies were resolved through discussion with a third investigator (GJJ).

**TABLE 4:** Evaluations of the bias risk and applicability of the included studies.

References	Risk of bias				Risk of applicability			Overall risk	
	Participants	Predictors	Outcome	Analysis	Participants	Predictors	Outcome	Risk of bias	Risk of applicability
Liu (25)	High	Unclear	Unclear	High	Low	Low	Low	High	Low
Wei (29)	Low	Low	Low	Low	Low	Low	Low	Low	Low
Long (26)	High	Low	Unclear	Unclear	Low	Low	Low	High	Low
Wang et al. (28)	High	Unclear	Unclear	Low	Low	Low	Low	High	Low
Lu et al. (35)	High	Unclear	High	Low	Low	Low	Low	High	Low
Xia Z et al. (37)	High	Unclear	Unclear	Low	Low	Low	Low	High	Low
Feng et al. (24)	High	Low	Unclear	High	Low	Low	Low	High	Low
Ma et al. (27)	High	Unclear	Unclear	High	Low	Low	Low	High	Low
Zhang et al. (32)	High	Unclear	Low	High	Low	Low	Low	High	Low
Li et al. (36)	High	Unclear	Unclear	Low	Low	Low	Low	High	Low
Zhong et al. (33)	High	Unclear	Low	Low	Low	Low	Low	High	Low
Zuo (39)	High	Unclear	Low	Low	Low	Low	Low	High	Low
Jiang et al. (34)	High	Unclear	High	Low	Low	Low	Low	High	Low
Zhong et al. (38)	High	Low	Low	Low	Low	Low	Low	High	Low
Dong (23)	High	Unclear	Unclear	Unclear	Low	Low	Low	High	Low
Yang et al. (30)	High	Unclear	Low	Low	Low	Low	Low	High	Low
Yin (31)	High	Unclear	Low	Low	Low	Low	Low	High	Low

High: high risk of bias or high applicability concern; Low: low risk of bias or low applicability concern; Unclear: unclear risk of bias or unclear applicability concern.

### 2.7 Data synthesis and statistical analysis

Study characteristics were summarized descriptively, including country, study design, sample source, sample size, participant characteristics, hypertension diagnostic criteria, cognitive assessment tool, and outcome prevalence. Model-level data included modeling method, candidate predictors, events per variable, variable-selection approach, handling of continuous variables, validation method, discrimination, calibration, model presentation, TRIPOD reporting, and final predictors. The frequency with which predictors were retained across final models was summarized descriptively. Model reproducibility was assessed in a separate table according to the availability of predictor definitions, regression coefficients or score assignments, intercepts, complete equations, nomograms, scoring rules, and implementation materials.

For the supplementary exploratory quantitative analysis, reported c-statistics (AUCs) were synthesized separately for derivation and validation datasets (22). Random-effects models were fitted using restricted maximum likelihood estimation in the metamisc package (valmeta) in R version 4.3.1. C-statistics were synthesized on the logit scale and back-transformed for presentation. When a 95% CI was reported, the corresponding standard error (SE) was derived from the reported confidence limits under a normal approximation. When a CI was unavailable, the SE was approximated from the reported c-statistic, total sample size (N), and number of participants classified with cognitive impairment (O) using the Newcombe asymptotic approach incorporating the modified Hanley–McNeil variance approximation, as implemented in valmeta. The detailed forest plots, subgroup analyses, and leave-one-model-out sensitivity analyses are reported in Supplementary Additional file 2, and a concise summary is provided at the end of the Results.

## 3 Results

### 3.1 Study selection

**FIGURE 1:**
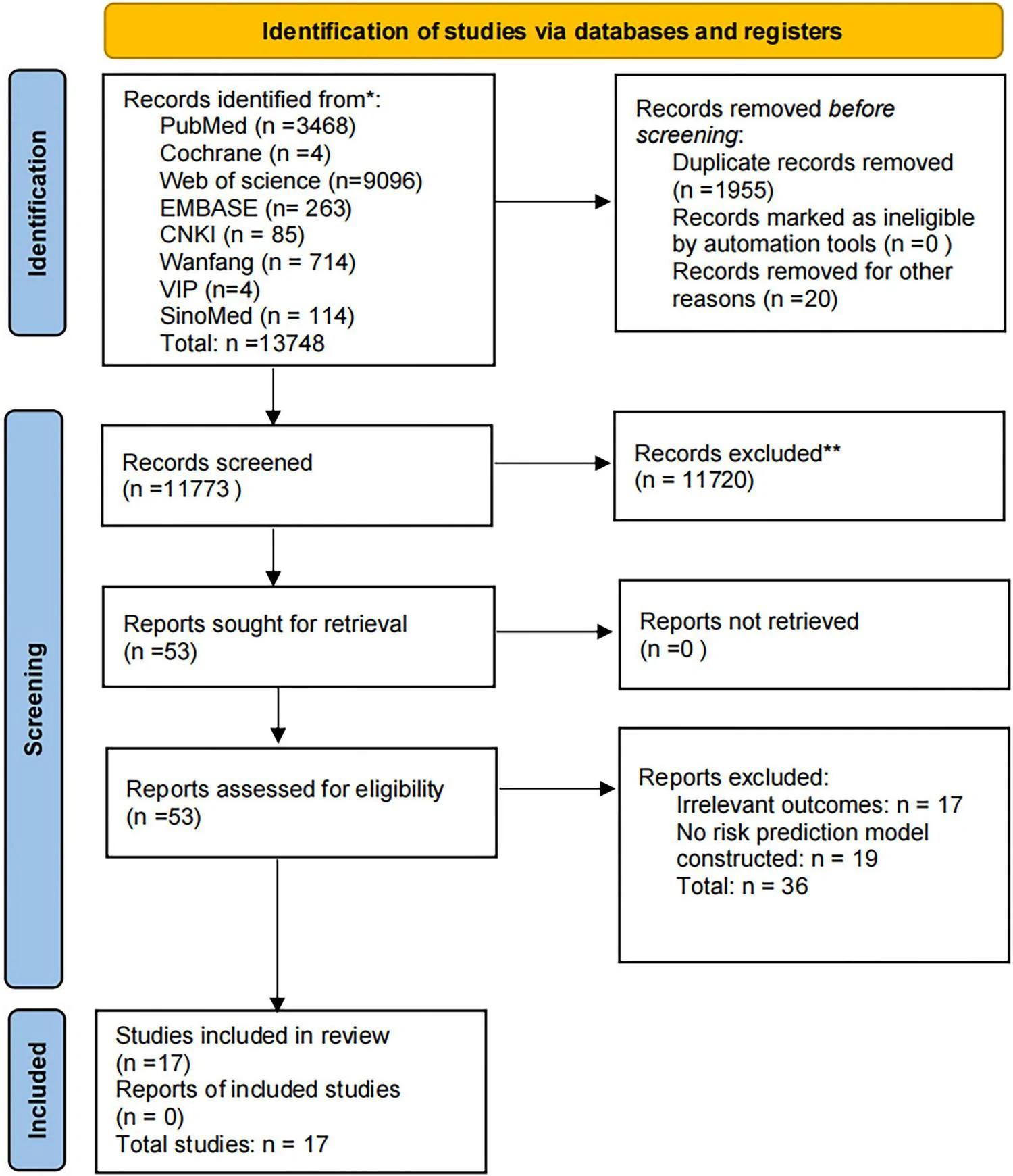
Flowchart of study selection.

The initial database search yielded 13,748 records. Following the screening process, 17 studies (23–39) were considered eligible for inclusion in the review. The full search strategies are reported in Supplementary Additional file 1, and the screening process with exclusion reasons is summarized in Figure 1.

### 3.2 Characteristics of the included studies

All 17 studies (23–39) were published between 2021 and 2025, including 11 (23–33) in Chinese and 6 (34–39) in English. With the exception of one study (39) utilizing the U.S. National Health and Nutrition Examination Survey (NHANES) database, all other studies (23–38) were conducted in China. Furthermore, except for one study (29) that employed a prospective cohort design, all other studies (23–28, 30–39) used a cross-sectional design. The total number of hypertensive patients included in the studies ranged from 137 to 6,024. The prevalence of mild cognitive impairment ranged from a minimum of 16.03% to a maximum of 68.13%. The primary cognitive function assessment tools used were the Mini-Mental State Examination (MMSE) and the Montreal Cognitive Assessment (MoCA). Other instruments included the Community Screening Instrument for Dementia, Consortium to Establish a Registry for Alzheimer’s Disease (CERAD) cognitive tests, Animal Fluency Test, Digit Symbol Substitution Test, Subjective Cognitive Decline Questionnaire-9, and China Health and Retirement Longitudinal Study (CHARLS) cognitive tests. These outcome constructs and assessment tools were described separately and were not considered interchangeable when interpreting model performance. The study participants were primarily hypertensive patients, covering a wide age range. Ten studies (23–26, 29, 32–35, 39) specifically analyzed elderly hypertensive patients. The diagnostic criteria for hypertension also varied. Information on antihypertensive treatment, blood pressure control status, and hypertension grade was inconsistently reported across studies and was therefore not harmonized as a common population characteristic. The sample sources for the studies were diverse, including inpatients, community populations, rural populations, etc. Among these, 3 (25, 29, 31) were dissertations, and 14 (23, 24, 26–28, 30, 32–39) were journal articles (Table 1).

### 3.3 Model establishment

The present analysis comprised 17 (23–39) articles, which together detailed 25 distinct prediction models. A variety of modeling techniques, variable selection strategies, and validation methods were employed across the studies, and the models exhibited heterogeneity in the number of candidate predictors, discrimination, and calibration. Notably, logistic regression was the primary modeling approach, with only one (36) exception. The number of candidate predictors considered for model development varied from 9 to 42. The most frequent variable selection strategy was a combination of univariate and multivariate analysis (*n* = 12) (23–29, 31, 32, 34, 35, 39), followed by LASSO regression for dimensionality reduction (*n* = 5) (30, 33, 36–38). One study (39) utilized a hybrid approach. Continuous variables were managed in one of two ways: most studies (*n* = 11) (24–26, 28, 29, 32, 33, 36–39) treated them as continuous (e.g., age, blood pressure values), whereas a smaller number of studies (*n* = 6) (23, 27, 30, 31, 34, 35) dichotomized or categorized them based on specific thresholds (Table 2).

### 3.4 Characteristics of models

Across the 17 studies (23–39), the AUC of the receiver operating characteristic ranged from 0.344 to 0.938. Two AUC values below 0.50, both reported in the Zhang 2023 study (32), reflected the discrimination of individual protective factors rather than complete multivariable models. Most multivariable models reported an AUC above 0.70. Lower AUCs were mainly observed in studies comparing several modeling approaches, including values of 0.535, 0.583, and 0.645 for less well-performing models (33, 38).

Internal validation, including bootstrap resampling, k-fold cross-validation, or split-sample validation, was reported in 15 studies (24, 25, 27–39). Four studies additionally reported external validation, including geographical or temporal validation (30, 34–36). Calibration was assessed in 11 studies using calibration curves, the Hosmer–Lemeshow goodness-of-fit test, or both. Three studies applied both approaches (34–36), whereas six studies did not report a calibration assessment (23, 25–27, 32, 33). Models were presented as nomograms in 10 studies (28–31, 33–37, 39), prediction formulas in three studies (24, 32, 36), and a risk score in one study (23). The presentation format of the remaining models was unclear.

**FIGURE 2:**
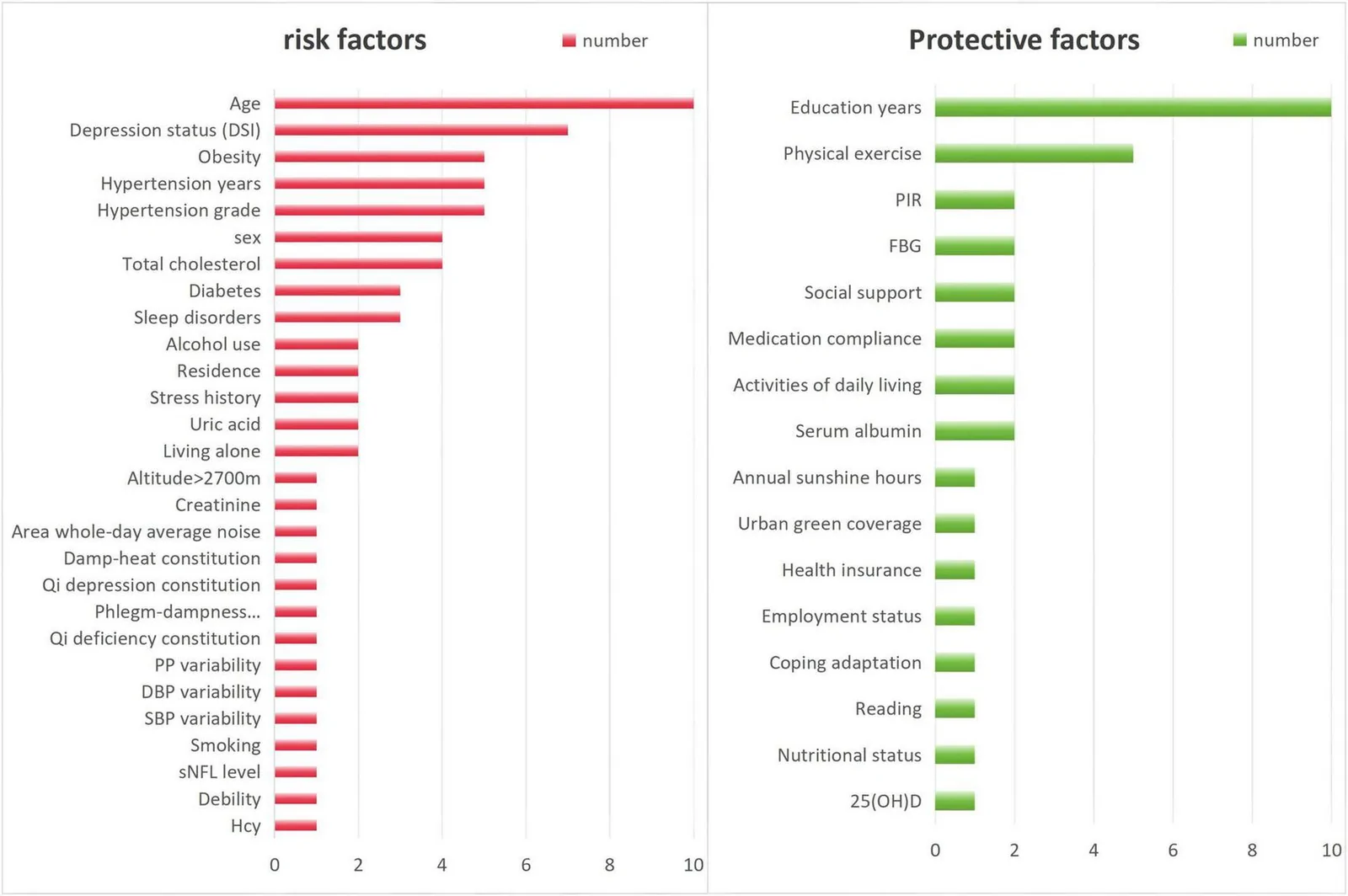
Predictor frequency chart.

Across the 17 studies, the final models included 3 to 11 predictors (Supplementary Additional file 3). Older age, depression, obesity, longer hypertension duration, higher hypertension grade, total cholesterol, and sex were frequently retained as risk-related predictors. Higher educational attainment and regular physical activity were more often retained as protective predictors (Figure 2).

### 3.5 Model reproducibility

Model reproducibility was limited. Four studies provided sufficient information for independent calculation of predicted probability or risk (24, 28, 32, 36). These studies reported complete equations, coefficients and intercepts, or sufficiently explicit formulas. Eight studies were partially reproducible because they provided nomograms, dynamic nomograms, risk scores, or coefficients without all components needed for exact reconstruction (23, 27, 29–31, 34, 35, 39). Five studies provided insufficient information for independent external validation (25, 26, 33, 37, 38). Machine-learning models were particularly limited because most did not provide code, trained model objects, tree structures, hyperparameters, or executable prediction tools. The reproducibility assessment is summarized in Table 3.

### 3.6 Exploratory quantitative analysis

The exploratory quantitative synthesis of c-statistics was reported in Supplementary Additional file 2. In derivation datasets, the pooled c-statistic was 0.83 (95% CI, 0.80–0.87; 95% prediction interval, 0.61–0.94). In validation datasets, the pooled c-statistic was 0.79 (95% CI, 0.76–0.81; 95% prediction interval, 0.68–0.87). These findings suggest that many models showed moderate-to-good discrimination in their original study contexts.

Logistic regression had a pooled c-statistic of 0.84 (95% CI, 0.80–0.87), whereas machine-learning estimates had very wide confidence intervals, including XGBoost (0.92; 95% CI, 0.20–1.00), AdaBoost (0.88; 95% CI, 0.18–1.00), Gaussian Naive Bayes (0.83; 95% CI, 0.093–1.00), and support vector machine (0.74; 95% CI, 0.089–1.00). Similarly, subgroup results by cognitive assessment tool were descriptive only: MMSE-based models showed a pooled c-statistic of 0.83 (95% CI, 0.78–0.87), and MoCA-based models showed a pooled c-statistic of 0.86 (95% CI, 0.60–0.96), but the MoCA prediction interval was extremely wide (0.0035–1.00).

Leave-one-model-out analyses showed limited estimate-level influence: the pooled c-statistic ranged from 0.82 to 0.84 for derivation datasets and from 0.79 to 0.80 for validation datasets. Nonetheless, these analyses could not account for within-study dependence because several studies contributed multiple models from the same dataset and covariance information was not reported. Accordingly, all quantitative findings are reported as supplementary exploratory analyses and should not be used to rank models, cognitive scales, or modeling approaches.

## 4 Quality assessment

A total of 17 studies (23–39) were included in this analysis. According to the PROBAST, only one study (29) was judged to have a low overall risk of bias; the remaining 16 studies (23–28, 30–39) were deemed to have a high risk. Assessment of risk of bias: Regarding the domain of participants, 16 studies (23–28, 30–39) were rated as high risk, chiefly because these studies employed a cross-sectional design, creating a potential for reverse causality as the outcome might have preceded the measurement of predictors. In the predictors domain, 13 studies (23, 25, 27, 28, 30–37, 39) failed to report if predictor assessment was conducted blinded to the outcome data, resulting in an unclear classification. Pertaining to the outcome domain, 7 studies (29–33, 38, 39) were classified as low risk because a reasonable time interval existed between the assessment of predictors and the ascertainment of the outcome. For the analysis domain, 6 studies (23–27, 32) were rated as high risk or unclear, primarily due to a lack of consideration for overfitting, underfitting, or optimal fit of the prediction models. The assessment of overall model applicability indicated a low risk of concerns regarding applicability for all included studies (Table 4).

## 5 Discussion

This systematic review and critical appraisal included 17 studies reporting 25 models for cognitive impairment among adults with hypertension. Reported c-statistics varied across models, and the supplementary random-effects meta-analysis was intended only as an exploratory descriptive summary. Given the predominance of cross-sectional designs, heterogeneous outcome definitions, variation in cognitive assessment tools, and multiple model estimates contributed by some datasets, pooled discrimination should not be interpreted as conclusive evidence of predictive utility. In particular, the subgroup summaries did not demonstrate superiority of machine-learning approaches over logistic regression. Across models, the most consistently retained predictors were older age, lower educational attainment, depressive symptoms, longer duration and greater severity of hypertension, and metabolic or biochemical indicators. Methodological appraisal indicated that most included models were at high risk of bias (16/17 by PROBAST), driven largely by cross-sectional designs, limited external validation, and incomplete calibration assessment.

Across the 25 models, higher predicted risk clustered in the following patient profiles: older individuals; those with lower educational attainment; patients with depressive symptoms; those with longer hypertension duration and/or higher hypertension grade; and, in multiple models, patients with obesity or adverse lipid profiles. A subset of models additionally incorporated serum biomarkers such as homocysteine and uric acid. The inclusion of these biomarkers in some models indicates potential predictive relevance; however, their incremental value beyond core clinical variables remains uncertain because comparative performance metrics were rarely reported.

The predominance of age, education, depression, and hypertension burden as recurrent model predictors aligns with established conceptual pathways for hypertension-related cognitive decline. Older age is a major determinant of neurodegeneration and reduced physiological reserve, and its strong association with cognitive impairment is consistently documented (40–42). Lower education, often interpreted as lower cognitive reserve, may reduce resilience to structural brain injury and neurodegenerative pathology, thereby increasing the likelihood that vascular insults translate into measurable impairment (43, 44). Depression may function both as a risk marker (reflecting psychosocial and health-behavior correlates) and as a biological contributor via hypothalamic–pituitary–adrenal axis dysregulation and neuroinflammatory signaling, mechanisms that are also implicated in cognitive deterioration (45–47). Finally, longer duration and greater severity of hypertension plausibly represent cumulative exposure to cerebrovascular dysregulation, blood–brain barrier injury, and cerebral small-vessel disease, which together contribute to vascular cognitive impairment (10, 48). Biomarkers such as homocysteine and uric acid, which were included in some models, are biologically plausible mechanistic correlates of oxidative stress and endothelial dysfunction. Their appearance as predictors suggests that models may gain from integrating vascular-metabolic injury signals; however, without standardized reporting of coefficients or model-agnostic importance, it remains unclear how much marginal predictive gain these biomarkers provide beyond core clinical variables (49, 50).

Reported discrimination estimates varied substantially across models. Although many models reported c-statistics above 0.70, these estimates were commonly obtained in derivation or internal-validation datasets and cannot be directly compared across heterogeneous outcomes or assessment tools. The supplementary leave-one-model-out analyses showed that the exploratory pooled summaries were not dominated by a single model estimate. The supplementary meta-analysis of c-statistics was undertaken only as an exploratory descriptive synthesis. Although several machine-learning approaches showed higher pooled point estimates than logistic regression, the confidence intervals for XGBoost, AdaBoost, GNB, and SVM were extremely wide and, in some instances, extended close to the lower and upper boundaries of the c-statistic scale. These imprecise estimates do not support claims that machine-learning models outperform logistic regression. Similarly, apparent differences between models based on MMSE and MoCA should not be interpreted as comparative evidence because these instruments assess related but non-interchangeable cognitive constructs. Accordingly, the pooled and subgroup findings should be considered supplementary summaries rather than evidence for selecting one modeling approach over another.

From a clinical standpoint, the convergence of predictors across diverse models suggests a pragmatic risk-stratification strategy for hypertensive care: prioritize cognitive screening and longitudinal monitoring in older adults, those with low education, depression, long-standing or more severe hypertension, and those with obesity or adverse lipid profiles, while recognizing that sex-related risk direction remains unclear in the current synthesis. These profiles map to readily obtainable clinical data, supporting feasibility for incorporation into routine follow-up and community hypertension management.

However, given the predominance of cross-sectional derivation studies and high risk of bias, model outputs should be used cautiously and primarily to enrich clinical judgment rather than to dictate management. In the near term, interpretable models built around consistently replicated predictors may be preferable for implementation and clinician trust. Future research should prioritize prospective cohorts with standardized cognitive outcomes and clear predictor-outcome temporality, rigorous external validation across regions and care settings, systematic calibration assessment and recalibration procedures, and transparent reporting aligned with TRIPOD to facilitate reproducibility and clinical translation.

To our knowledge, this is the first study to systematically map and critically appraise models intended to identify cognitive impairment in patients with hypertension. The review suggests that existing models may help distinguish hypertensive patients with and without cognitive impairment in their development settings, but evidence supporting future-risk prediction and comparative superiority of particular modeling approaches remains limited. Clinicians should consider the development population, outcome definition, validation status, calibration, and reproducibility before using any model. Future efforts should shift from developing new models to conducting rigorous prospective validation, methodological optimization, and clinical impact assessment of existing promising models, ultimately realizing their practical value in managing the cognitive health of hypertensive patients.

## Data Availability

The original contributions presented in this study are included in the article/Supplementary material, further inquiries can be directed to the corresponding authors.
