# Supplementary material for: Identification of cognitive impairment in patients with hypertension: a systematic review and critical appraisal of existing models
Source: Front Med (Lausanne). 2026 Aug 10;13:1858790. doi: 10.3389/fmed.2026.1858790 (PMC13500545; doi:10.3389/fmed.2026.1858790)
Supplement: Supplementary file 1 [file Supplementary_file_1.docx]

**Search strategies**

**Table S1 PubMed: 3468 records.**

| #1 | "Hypertension"[Mesh] |
| --- | --- |
| #2 | ((((((Hypertension[Title/Abstract])) OR (Blood Pressure, High[Title/Abstract])) OR (Blood Pressures, High[Title/Abstract])) OR (High Blood Pressure[Title/Abstract])) OR (High Blood Pressures[Title/Abstract])) OR (HTN[Title/Abstract]) |
| #3 | #1 OR #2 |
| #4 | "Neurocognitive Disorders"[Mesh] OR "Cognitive Dysfunction"[Mesh] OR "Dementia"[Mesh] |
| #5 | (((((((((((((((((((((((((((((((((((((((Neurocognitive Disorders[Title/Abstract])) OR (Cognitive Dysfunction[Title/Abstract])) OR (Dementia[Title/Abstract])) OR (Disorder*, Neurocognitive[Title/Abstract])) OR (Neurocognitive Disorder[Title/Abstract])) OR (Kandinsky Syndrome[Title/Abstract])) OR (Clerambault Syndrome[Title/Abstract])) OR (Organic Mental Disorders, Psychotic[Title/Abstract])) OR (Delirium, Dementia, Amnestic, Cognitive Disorders[Title/Abstract])) OR (Disorders, Organic Mental[Title/Abstract])) OR (Mental Disorder*, Organic[Title/Abstract])) OR (Organic Mental Disorder*[Title/Abstract])) OR (Mild Neurocognitive Disorder*[Title/Abstract])) OR (Disorder*, Mild Neurocognitive[Title/Abstract])) OR (Neurocognitive Disorder*, Mild[Title/Abstract])) OR (Organic Brain Syndrome, Nonpsychotic[Title/Abstract])) OR (Nonpsychotic Organic Brain Syndrome[Title/Abstract])) OR (Psychoses, Traumatic[Title/Abstract])) OR (Traumatic Psychoses[Title/Abstract])) OR (Cognitive Dysfunctions[Title/Abstract])) OR (Dysfunction*, Cognitive[Title/Abstract])) OR (Cognitive Impairment*[Title/Abstract])) OR (Impairment*, Cognitive[Title/Abstract])) OR (Cognitive Disorder*[Title/Abstract])) OR (Disorder*, Cognitive[Title/Abstract])) OR (Mild Cognitive Impairment*[Title/Abstract])) OR (Cognitive Impairment*, Mild[Title/Abstract])) OR (Impairment*, Mild Cognitive[Title/Abstract])) OR (Cognitive Decline*[Title/Abstract])) OR (Decline*, Cognitive[Title/Abstract])) OR (Mental Deterioration*[Title/Abstract])) OR (Deterioration*, Mental[Title/Abstract])) OR (Dementias[Title/Abstract])) OR (Amentia*[Title/Abstract])) OR (Senile Paranoid Dementia*[Title/Abstract])) OR (Dementias, Senile Paranoid[Title/Abstract])) OR (Paranoid Dementia*, Senile[Title/Abstract])) OR (Familial Dementia*[Title/Abstract])) OR (Dementia*, Familial[Title/Abstract]) |
| #6 | #4 OR #5 |
| #7 | "Predictive Learning Models"[Mesh] |
| #8 | ((((((((((((((((((((Predictive Learning Models[Title/Abstract])) OR (Prediction Model[Title/Abstract])) OR (Predictive Model[Title/Abstract])) OR (Predictive Modeling[Title/Abstract])) OR (Prognostic Model[Title/Abstract])) OR (Machine Learning Model[Title/Abstract])) OR (Risk Model[Title/Abstract])) OR (Risk Prediction Model[Title/Abstract])) OR (Risk Assessment Model[Transliterated Title])) OR (Risk Forecast Model[Title/Abstract])) OR (Prediction Tool[Title/Abstract])) OR (Predictive Tool[Title/Abstract])) OR (Risk Assessment[Title/Abstract])) OR (Risk Calculation[Title/Abstract])) OR (Risk Factors[Title/Abstract])) OR (Risk Prediction[Title/Abstract])) OR (Risk Score[Title/Abstract])) OR (Nomogram[Title/Abstract])) OR (Scoring System[Title/Abstract])) OR (Models, Statistical[Title/Abstract]) |
| #9 | #7 OR #8 |
| #10 | #3 AND #6 AND #9 |

**Table S2. The Cochrane library: 4 records.**

| #1 | MeSH descriptor: [Hypertension] explode all trees |
| --- | --- |
| #2 | (Hypertension):ti,ab,kw OR (Blood Pressures, High):ti,ab,kw OR (High Blood Pressure):ti,ab,kw OR (High Blood Pressures):ti,ab,kw OR (Blood Pressure, High):ti,ab,kw |
| #3 | #1 OR #2 |
| #4 | MeSH descriptor: [Neurocognitive Disorders] explode all trees |
| #5 | (Neurocognitive Disorders):ti,ab,kw OR (Organic Mental Disorders, Psychotic):ti,ab,kw OR (Nonpsychotic Organic Brain Syndrome):ti,ab,kw OR (Organic Brain Syndrome, Nonpsychotic):ti,ab,kw OR (Clerambault Syndrome):ti,ab,kw |
| #6 | (Kandinsky Syndrome):ti,ab,kw OR (Disorder, Neurocognitive):ti,ab,kw OR (Neurocognitive Disorder; Disorders, Neurocognitive):ti,ab,kw OR (Psychoses, Traumatic):ti,ab,kw OR (Traumatic Psychoses):ti,ab,kw |
| #7 | (Disorders, Organic Mental):ti,ab,kw OR (Delirium, Dementia, Amnestic, Cognitive Disorders):ti,ab,kw OR (Organic Mental Disorders):ti,ab,kw OR (Mental Disorder, Organic):ti,ab,kw OR (Mental Disorders, Organic):ti,ab,kw |
| #8 | (Organic Mental Disorder):ti,ab,kw OR (Disorder, Mild Neurocognitive):ti,ab,kw OR (Mild Neurocognitive Disorder):ti,ab,kw OR (Disorders, Mild Neurocognitive):ti,ab,kw OR (Neurocognitive Disorder, Mild):ti,ab,kw |
| #9 | (Mild Neurocognitive Disorders):ti,ab,kw OR (Neurocognitive Disorders, Mild):ti,ab,kw |
| #10 | #4 OR #5 OR #6 OR #7 OR #8 OR #9 |
| #11 | MeSH descriptor: [Predictive Learning Models] explode all trees |
| #1 | (Predictive Learning Models):ti,ab,kw OR (Machine Predictive Models):ti,ab,kw OR (Model, Machine Predictive):ti,ab,kw OR (Learning Model, Predictive):ti,ab,kw OR (Model, Predictive Learning):ti,ab,kw |
| #13 | (Machine Predictive Model):ti,ab,kw OR (Predictive Model, Machine):ti,ab,kw OR (Predictive Learning Model):ti,ab,kw OR (Predictive Models, Machine):ti,ab,kw |
| #14 | #11 OR #12 OR #13 |
| #15 | #3 AND #10 AND #14 |

**Table S3. Web of science: 9096 records.**

| #1 | TS=(Hypertension OR Blood Pressure, High OR Blood Pressures, High OR High Blood Pressure OR High Blood Pressures OR hypertensive patient*) |
| --- | --- |
| #2 | TS=(Neurocognitive Disorders OR Cognitive Dysfunction OR Dementia OR Disorder*, Neurocognitive OR Neurocognitive Disorder OR Kandinsky Syndrome OR Clerambault Syndrome OR Organic Mental Disorders, Psychotic OR Delirium, Dementia, Amnestic, Cognitive Disorders OR Disorders, Organic Mental OR Mental Disorder*, Organic OR Organic Mental Disorder* OR Mild Neurocognitive Disorder* OR Disorder*, Mild Neurocognitive OR Neurocognitive Disorder*, Mild OR Organic Brain Syndrome, Nonpsychotic OR Nonpsychotic Organic Brain Syndrome OR Psychoses, Traumatic OR Traumatic Psychoses OR Cognitive Dysfunctions OR Dysfunction*, Cognitive OR Cognitive Impairment* OR Impairment*, Cognitive OR Cognitive Disorder* OR Disorder*, Cognitive OR Mild Cognitive Impairment* OR Cognitive Impairment*, Mild OR Impairment*, Mild Cognitive OR Cognitive Decline* OR Decline*, Cognitive OR Mental Deterioration* OR Deterioration*, Mental OR Dementias OR Amentia* OR Senile Paranoid Dementia* OR Dementias, Senile Paranoid OR Paranoid Dementia*, Senile OR Familial Dementia* OR Dementia*, Familial) |
| #3 | TS=(Predictive Learning Models OR Prediction Model OR Predictive Model OR Predictive Modeling OR Prognostic Model OR Machine Learning Model OR Risk Model OR Risk Prediction Model OR Risk Assessment Model[Transliterated Title] OR Risk Forecast Model OR Prediction Tool OR Predictive Tool OR Risk Assessment OR Risk Calculation OR Risk Factors OR Risk Prediction OR Risk Score OR Nomogram OR Scoring System OR Models, Statistical) |
| #4 | #1 AND #2 AND #3 |

**Table S4. EMBASE: 263records.**

| #1 | 'hypertension'/exp |
| --- | --- |
| #2 | hypertension:ab,ti OR 'acute hypertension':ab,ti OR 'arterial hypertension':ab,ti OR 'blood pressure, high':ab,ti OR 'cardiovascular hypertension':ab,ti OR 'controlled hypertension':ab,ti OR 'endocrine hypertension':ab,ti OR 'high blood pressure':ab,ti OR 'high renin hypertension':ab,ti OR (htn:ab,ti AND hypertension:ab,ti) OR 'hypertensive disease':ab,ti OR 'hypertensive effect':ab,ti OR 'hypertensive reaction':ab,ti OR 'hypertensive response':ab,ti OR 'neurogenic hypertension':ab,ti OR 'preexistent hypertension':ab,ti OR 'salt high blood pressure':ab,ti OR 'salt hypertension':ab,ti OR 'secondary hypertension':ab,ti OR 'systemic hypertension':ab,ti |
| #3 | #1 OR #2 |
| #4 | 'cognitive defect'/exp |
| #5 | 'cognition disorder':ab,ti OR 'cognition disorders':ab,ti OR 'cognitive complaints':ab,ti OR 'cognitive decline':ab,ti OR 'cognitive defects':ab,ti OR 'cognitive deficiency':ab,ti OR 'cognitive deficit':ab,ti OR 'cognitive difficulties':ab,ti OR 'cognitive disability':ab,ti OR 'cognitive disorder':ab,ti OR 'cognitive disorders':ab,ti OR 'cognitive disturbance':ab,ti OR 'cognitive dysfunction':ab,ti OR 'cognitive impairment':ab,ti OR 'cognitive problems':ab,ti OR 'delirium, dementia, amnestic, cognitive disorders':ab,ti OR overinclusion:ab,ti OR 'response interference':ab,ti OR 'cognitive defect':ab,ti |
| #6 | #4 OR #5 |
| #7 | 'predictive model'/exp |
| #8 | 'forecast model':ab,ti OR 'forecast modeling':ab,ti OR 'forecast modelling':ab,ti OR 'forecast simulation':ab,ti OR 'forecasting model':ab,ti OR 'prediction model':ab,ti OR 'predictive modeling':ab,ti OR 'predictive modelling':ab,ti OR 'predictive simulation':ab,ti OR 'predictive model':ab,ti |
| #9 | #7 OR #8 |
| #10 | #3 AND #6 AND #9 |

**Table S5. CNKI: 85 records.**

| #1 | 主题=(高血压) |
| --- | --- |
| #2 | 主题=(神经认知障碍 OR 认知功能障碍 OR 认知功能损害 OR 认知衰退 OR 认知功能下降 OR 痴呆 OR 血管性认知障碍 OR 血管性痴呆 OR 轻度认知障碍 OR MCI) |
| #3 | 主题=(预测模型 OR 风险预测模型 OR 风险评分 OR 列线图 OR Nomogram OR 预测工具 OR 预测因子 OR 预后模型) |
| #4 | #1 AND #2 AND #3 |

**Table S6. Wanfang: 714 records.**

| #1 | 主题=(高血压) |
| --- | --- |
| #2 | 主题=(神经认知障碍 OR 认知功能障碍 OR 认知功能损害 OR 认知衰退 OR 认知功能下降 OR 痴呆 OR 血管性认知障碍 OR 血管性痴呆 OR 轻度认知障碍 OR MCI) |
| #3 | 主题=(预测模型 OR 风险预测模型 OR 风险评分 OR 列线图 OR Nomogram OR 预测工具 OR 预测因子 OR 预后模型) |
| #4 | #1 AND #2 AND #3 |

**Table S7. VIP: 4 records.**

| #1 | M=高血压 |
| --- | --- |
| #2 | (K=神经认知障碍 OR K=认知功能障碍 OR K=认知功能损害 OR K=认知衰退 OR K=轻度认知障碍 OR K=认知功能下降 OR K=痴呆 OR K=血管性认知障碍 OR K=血管性痴呆 OR K=轻度认知障碍 OR K=MCI) |
| #3 | (K=预测模型 OR K=风险预测模型 OR K=风险评分 OR K=列线图 OR K=Nomogram OR K=预测工具 OR K=预测因子 OR K=预后模型) |
| #4 | #1 AND #2 AND #3 |

**Table S8. SinoMed: 114 records.**

| #1 | (高血压) |
| --- | --- |
| #2 | (神经认知障碍 OR 认知功能障碍 OR 认知功能损害 OR 认知衰退 OR 认知功能下降 OR 痴呆 OR 血管性认知障碍 OR 血管性痴呆 OR 轻度认知障碍 OR MCI) |
| #3 | (预测模型 OR 风险预测模型 OR 风险评分 OR 列线图 OR Nomogram OR 预测工具 OR 预测因子 OR 预后模型) |
| #4 | #1 AND #2 AND #3 |
