# Supplementary material for: Identification of cognitive impairment in patients with hypertension: a systematic review and critical appraisal of existing models
Source: Front Med (Lausanne). 2026 Aug 10;13:1858790. doi: 10.3389/fmed.2026.1858790 (PMC13500545; doi:10.3389/fmed.2026.1858790)
Supplement: Supplementary file 2 [file Supplementary_file_2.docx]

**Supplementary exploratory meta-analysis of c-statistics**

**Results of the training set Models**

In the training datasets **(Figure 3)**, the pooled c-statistic was 0.83 (95% CI, 0.80–0.87), indicating good overall discrimination. Although between-study heterogeneity was present, the 95% prediction interval remained above the no-discrimination threshold of 0.50 (0.61–0.94) and was directionally consistent with the pooled CI, suggesting that heterogeneity did not materially affect the overall conclusion.

Figure 3. Forest plot of the training set models

**Results of the validation set Models**

In the validation datasets **(Figure 4)**, the pooled c-statistic was 0.79 (95% CI, 0.76–0.81), indicating overall good discrimination. The 95% prediction interval also remained above 0.50 (0.68–0.87) and was consistent with the pooled CI, suggesting that the observed heterogeneity was unlikely to change the interpretation of the overall validation performance.

Figure 4. Forest plot of the validation set models

**Exploratory subgroup analysis**

**Modeling method**

**Figure 5** presents exploratory subgroup summaries by modelling approach. Logistic regression had a pooled c-statistic of 0.84 (95% CI, 0.80–0.87). The corresponding pooled point estimates were 0.92 (95% CI, 0.20–1.00) for XGBoost, 0.88 (95% CI, 0.18–1.00) for AdaBoost, 0.83 (95% CI, 0.093–1.00) for GNB, and 0.74 (95% CI, 0.089–1.00) for SVM. However, the confidence intervals for the machine-learning approaches were extremely wide, with some spanning values close to no discrimination and nearly perfect discrimination. These estimates are too imprecise to establish that any machine-learning approach has better discrimination than logistic regression. No formal between-subgroup comparison was performed, and the results should not be used to rank modelling approaches.

Figure 5. Subgroup forest plot of modeling approaches. (A) Logistic Regression (LR); (B) Gaussian Naive Bayes (GNB); (C) Support Vector Machines (SVM); (D) Extreme Gradient Boosting (XGB); (E) Adaptive Boosting (AdaBoost)

**Cognitive assessment scale**

In the exploratory subgroup analysis by cognitive assessment tool **(Figure 6)**, MMSE-based models showed a pooled c-statistic of 0.83 (95% CI, 0.78–0.87), and the corresponding 95% prediction interval did not cross 0.50, suggesting that between-study heterogeneity did not materially affect the interpretation of this subgroup result. MoCA-based models showed a pooled c-statistic of 0.86 (95% CI, 0.60–0.96). However, the 95% prediction interval for the MoCA subgroup ranged from 0.0035 to 1.00, crossing 0.50 and indicating uncertainty related to between-study heterogeneity. Therefore, this subgroup result should be interpreted with caution.

Figure 6. Subgroup forest plot of different scales. (A) Mini-Mental State Examination (MMSE); (B) Montreal Cognitive Assessment (MoCA)

**Sensitivity analysis**

For derivation datasets, leave-one-model-out sensitivity analysis **(Figure 7)** was performed by sequentially omitting each model estimate. The pooled c-statistic changed little, ranging from 0.82 to 0.84, and no single model estimate materially changed the summary result. This analysis evaluates the influence of individual model estimates but does not remove or formally model correlation among estimates derived from the same dataset.

Figure 7. Sensitivity analysis of training set

For validation datasets, leave-one-model-out sensitivity analysis **(Figure 8)** was performed by sequentially omitting each model estimate. The pooled c-statistic remained between 0.79 and 0.80, and no individual model estimate materially altered the summary result. This analysis evaluates estimate-level influence only and does not resolve within-study dependence.

Figure 8. Sensitivity analysis of validation set

**Legends**

Figure. 3 Forest plot of the training set models

Figure. 4 Forest plot of the validation set models

Figure. 5 Subgroup forest plot of modeling approaches. (A) Logistic Regression (LR); (B) Gaussian Naive Bayes (GNB); (C) Support Vector Machines (SVM); (D) Extreme Gradient Boosting (XGB); (E) Adaptive Boosting (AdaBoost)

Figure. 6 Subgroup forest plot of different scales. (A) Mini-Mental State Examination (MMSE); (B) Montreal Cognitive Assessment (MoCA)

Figure. 7 Sensitivity analysis of training set

Figure. 8 Sensitivity analysis of validation set
