# Supplementary material for: Identification of cognitive impairment in patients with hypertension: a systematic review and critical appraisal of existing models
Source: Front Med (Lausanne). 2026 Aug 10;13:1858790. doi: 10.3389/fmed.2026.1858790 (PMC13500545; doi:10.3389/fmed.2026.1858790)
Supplement: Supplementary file 3 [file Supplementary_file_3.docx]

| Study | Predictors |
| --- | --- |
| Liu (2021)  Wei (2021)  Long (2022)  Wang (2022)  Lu (2023)  Xia (2023)  Feng (2023)  Ma (2023)  Zhang (2023)  Li (2024)  Zhong (2024)  Zuo (2025)  Jiang (2025)  Zhong (2025)  Dong(2025)  Yang (2025)  Yin (2025) | (5 factors): Depression status (DSI), Age, Total cholesterol, Activities of daily living, Serum albumin.  (3 factors): systolic blood pressure (SBP) variability, diastolic blood pressure (DBP) variability, pulse pressure (PP) variability.  (5 factors): Age, education level, serum neurofilament light chain protein (sNFL) level, MMSE score, MoCA score.  (5 factors): Age,Diabetes, Daily life management, Education years, Coping adaptation.  (11 factors): Hypertension grade, Education level, Diabetes, Hypertension years, Stress history, Smoking, Physical exercise, Reading, Social support, Sleep disorders, Medication compliance.  (4 factors): Hip circumference, Age, Education level, Physical activity.  (4 factors): Hypertension duration, homocysteine(Hcy), 25 (OH) D, Uric acid.  (6 factors): Age ≥ 75 years, Female sex, Albumin level, Depression score, Hypertension grade 2, Hypertension grade 3.  (5 factors): Education years, fasting blood glucose (FBG), high blood pressure (HBP) duration, serum uric acid (SUA), total cholesterol (TC).  (8 factors): Gender, Age, Residence, Education, Alcohol use, Depression, Employment status, Health insurance.  (8 factors): Waist-hip ratio, Qi depression constitution, Age, Total cholesterol, Phlegm-dampness constitution, Damp-heat constitution, Qi deficiency constitution, Fasting blood glucose.  (7 factors): Sex, Age, Education, poverty-to-income ratio (PIR), Depression, Vigorous work activity, Creatinine.  (8 factors): Education level, Exercise, Hypertension duration, Blood pressure grade, Depression, Nutritional status, Debility, Sleep disorders.  (6 factors): Age, Waist circumference, Urban green coverage, Educational levels, Annual sunshine hours, Area whole-day average noise.  (10 factors): Living alone, Alcohol consumption, Diabetes, Sleep disorders, Hypertension stage II-III, Hypertension duration ≥ 5 years, Poor medication adherence, Low SSRS score (≤ 35), Stress history, Depression.  (8 factors): Age, Sex, BMI, Education, Residence, Physical activity, Depression, Total cholesterol.  (7 factors): Altitude > 2700m, Age ≥ 65 years, Obesity (BMI ≥ 28.0 kg/m²), Non-married status, Low education level (Primary school or below), Low income (< 20,000 CNY/year), Poor blood pressure control. |

Additional file 3. Final predictors for all models.
